# Suppression of the cough reflex by *α*_2_-adrenergic receptor agonists in the rabbit

**DOI:** 10.1002/phy2.122

**Published:** 2013-11-05

**Authors:** Elenia Cinelli, Fulvia Bongianni, Tito Pantaleo, Donatella Mutolo

**Affiliations:** Dipartimento di Medicina Sperimentale e Clinica, Sezione Scienze Fisiologiche, Università degli Studi di FirenzeViale G.B. Morgagni 63, 50134, Firenze, Italy

**Keywords:** Caudal nucleus tractus solitarii, caudal ventral respiratory group, clonidine, cough reflex, tizanidine, yohimbine

## Abstract

The *α*_2_-adrenergic receptor agonist clonidine has been shown to inhibit citric acid-induced cough responses in guinea pigs when administered by aerosol, but not orally. In contrast, oral or inhaled clonidine had no effect on capsaicin-induced cough and reflex bronchoconstriction in humans. In addition, intravenous administration of clonidine has been shown to depress fentanyl-induced cough in humans. We investigated the effects of the *α*_2_-adrenergic receptor agonists, clonidine and tizanidine, on cough responses induced by mechanical and chemical (citric acid) stimulation of the tracheobronchial tree. Drugs were microinjected (30–50 nL) into the caudal nucleus tractus solitarii (cNTS) and the caudal ventral respiratory group (cVRG) as well as administered intravenously in pentobarbital sodium-anesthetized, spontaneously breathing rabbits. Bilateral microinjections of clonidine into the cNTS or the cVRG reduced cough responses at 0.5 mmol/L and abolished the cough reflex at 5 mmol/L. Bilateral microinjections of 0.5 mmol/L tizanidine into the cNTS completely suppressed cough responses, whereas bilateral microinjections of 5 mmol/L into the cVRG only caused mild reductions in them. Depressant effects on the cough reflex of clonidine and tizanidine were completely reverted by microinjections of 10 mmol/L yohimbine. Intravenous administration of clonidine (80–120 μg/kg) or tizanidine (150–300 μg/kg) strongly reduced or completely suppressed cough responses. These effects were reverted by intravenous administration of yohimbine (300 μg/kg). The results demonstrate that activation of *α*_2_-adrenergic receptors in the rabbit exerts potent inhibitory effects on the central mechanism generating the cough motor pattern with a clear action at the level of the cNTS and the cVRG.

## Introduction

Cough is one of the most important airway defensive reflex (Korpáš and Tomori 1979) that involves several brainstem structures (Kubin and Davies 1995; Bongianni et al. 2005; Mazzone et al. 2005; Bolser et al. 2006; Bonham et al. 2006; Kubin et al. 2006; Mutolo et al. 2007, 2008b, 2009, 2010, 2012; Poliacek et al. 2007, 2011; Canning and Mori 2010, 2011) including the first and the last relay medullary station of the reflex pathway, that is, the second-order neurons within the caudal aspect of the nucleus tractus solitarii (cNTS) and the expiratory premotor neurons of the caudal ventral respiratory group (cVRG). It is widely agreed that tracheobronchial rapidly adapting receptors (RARs) are involved in cough mediation. They are responsive not only to mechanical stimulation but also to other types of stimuli including acid solutions. On the other hand, the role of bronchopulmonary C-fibers and A*δ*-nociceptive pulmonary afferent fibers in this reflex is controversial (see, e.g., Sant'Ambrogio and Widdicombe 2001; for further references see Mutolo et al. 2007, 2009, 2012). Recently, it has also been provided evidence that a receptor subtype found in the larynx and rostral trachea, quite distinct from the well-defined slowly adapting stretch receptors (SARs) and RARs, is primarily involved in the mediation of cough in guinea pigs (Canning et al. 2004). These receptors, termed “cough receptors”, are innervated by slow-conducting A*δ*-fibers. They are activated by punctate mechanical stimulation and acid, but are unresponsive to capsaicin, bradykinin, smooth muscle contraction, longitudinal or transverse stretching of the airways, or lung distension. For other details on the characteristics of receptors involved in cough, (see, e.g., Mutolo et al. 2007, 2009 and Sant'Ambrogio and Widdicombe 2001).

Reflex cough is purposeful under many circumstances (appropriate cough), but is without an apparent aim (inappropriate cough) in case of chronic cough (Haque et al. 2005). Antitussive drugs possess little clinically relevant efficacy and their use is limited by important side effects (Barnes 2007). A better understanding of neural mechanisms involved in acute and chronic cough could lead to more effective antitussive treatments.

Kubin et al. (2006) in a review on central pathways of pulmonary and lower airway vagal afferents suggested that receptors commonly associated with presynaptic effects, such as *α*_2_-adrenergic receptors, may affect respiratory reflexes. We reasoned that the latter may include the cough reflex. The *α*_2_-adrenergic receptor agonist clonidine has been shown to inhibit citric acid-induced cough responses in guinea pigs when administered by aerosol, but not orally (O'Connell et al. 1994). In contrast, oral or inhaled clonidine had no effect on capsaicin-induced cough and reflex bronchoconstriction in healthy humans (O'Connell et al. 1994). The antitussive effect of inhaled clonidine must have occurred via a local action in the airways, suggesting that there are inhibitory *α*_2_-adrenergic receptors on sensory nerves that mediate cough in the guinea pig. In addition, pretreatment with intravenous administration of clonidine has been shown to suppress fentanyl-induced cough in humans with mild hemodynamic changes (Horng et al. 2007).

Although clonidine is an *α*_2_-adrenergic receptor agonist prescribed historically as a centrally acting antihypertensive agent, new uses have been described (Eisenach et al. 1996; Philipp et al. 2002; Pertovaara 2006). Several clinical studies demonstrated the efficacy of intrathecal clonidine for the treatment of postoperative, neuropathic or cancer-associated pain (see Chan et al. 2010 for reviews). Intrathecal administration of *α*_2_-adrenergic receptor agonists, clonidine and tizanidine, synergistically interacts with lidocaine to enhance the degree of antinociception to somatic noxious stimuli in rats (Kawamata et al. 1997) and reduces experimental thermal- or capsaicin-induced pain and hyperalgesia in normal volunteers (Eisenach et al. 1998).

Much of what is known about airway cough-related pathways correlates well with studies on somatic reflex pathways, particularly those involved in nociception. Peripheral and central mechanisms subserving nociception and cough share similar features (e.g., Barnes 2007; Canning 2009; Ji et al. 2009; Mutolo et al. 2012; Lavinka and Dong 2013), such as the type of afferent fibers (A*δ* and C), transient receptor potential vanilloid 1, and transient receptor potential ankyrin 1 channels, as well as central and peripheral sensitization and the involvement of mitogen-activated protein kinase (MAPK) signal transduction pathways. In addition, there is a general agreement that the NTS is a possible target for synaptic plasticity and a strategic site where cough-related sensory inputs can be modulated (e.g., Mazzone et al. 2005; Bonham et al. 2006; Mutolo et al. 2007, 2008b; Canning and Mori 2011; Woolf 2011; see Mutolo et al. 2012 also for further references). Owing to the similarities between the characteristics of central processing of nociceptive and cough-related inputs, we reasoned that *α*_2_-adrenergic receptor activation could also have a role in the modulation of the cough reflex at the central level. Thus, we investigated the effects of clonidine and tizanidine microinjected into the cNTS and the cVRG as well as administered intravenously on cough responses induced by mechanical and chemical stimulation of the tracheobronchial tree in pentobarbital sodium-anesthetized, spontaneously breathing rabbits.

The results of different reports (Macron et al. 1994; Dutschmann et al. 1998; Plevkova et al. 2009) suggest that the cNTS and convergent inputs from nasal and vagal afferents may have a role in the regulation of nasotrigeminal reflex responses. Thus, an attempt was made to ascertain whether the sneeze reflex, a defensive motor act that shares many common features with the cough reflex (Korpáš and Tomori 1979; Shannon et al. 1997), was affected by clonidine or tizanidine. In addition, the cVRG is also the last medullary station of the sneeze reflex (Korpáš and Tomori 1979; Jakuš et al. 1985; Shannon et al. 1997).

## Material and Methods

### Animal preparation

Experiments were performed on 37 male New Zealand white rabbits (2.7–3.4 kg) anesthetized with pentobarbital sodium (40 mg/kg intravenous, supplemented by 2–4 mg/kg every 30 min; Sigma-Aldrich, St. Louis, MO). Atropine (0.15 mg/kg intramuscular) was administered to reduce mucosal secretion in the airways. The adequacy of anesthesia was assessed by the absence of reflex withdrawal of the hind limb in response to noxious pinching of the hind paw. Additional criteria were the presence of a stable and regular pattern of phrenic bursts and the absence of fluctuations in arterial blood pressure or phrenic nerve activity, whether spontaneous or in response to somatic nociceptive stimulation. All animal care and experimental procedures were conducted in accordance with the Italian legislation and the official regulations of the European Community Council on the use of laboratory animals (Directive 86/609/EEC). The study was approved by the Animal Care and Use Committee of the University of Florence. All efforts were made to minimize both the number of animals used and their suffering.

Experimental procedures and details about the methods employed have previously been described (Bongianni et al. 2005; Mutolo et al. 2007, 2008a,b, 2009, 2010, 2012; Cinelli et al. 2012). After cannulation of the trachea, polyethylene catheters were inserted into a femoral artery and vein for monitoring arterial blood pressure and for drug delivery, respectively. The C_3_ or C_5_ phrenic root was dissected free, cut distally, and prepared for recordings. The animal was placed in a prone position and fixed by a stereotaxic head holder and vertebral clamps; the head was ventroflexed for optimal exposure of the dorsal surface of the medulla by occipital craniotomy. Body temperature was maintained at 38.5–39°C by a heating blanket controlled by a rectal thermistor probe.

### Recording procedures

Efferent phrenic nerve activity was recorded using bipolar platinum electrodes from the central stump of the cut and desheathed C_3_ or C_5_ phrenic root. The electromyographic (EMG) activity of abdominal muscles was recorded by wire electrodes (Nichrome wires, insulated except for 1 mm at the tips, diameter 0.1 mm) inserted into the external or the internal oblique abdominal muscles. Phrenic and abdominal activities were amplified, full-wave rectified, and “integrated” (low-pass RC filter, time constant 100 msec). Extracellular recordings from medullary neurons were performed with tungsten microelectrodes (5–10 MΩ impedance at 1 kHz). The most rostral extent of the area postrema on the midline was defined as the obex and used as a reference point. Neuronal activity was recorded from expiratory neurons of the cVRG (1.6–3.0 mm caudal to the obex, 2.0–2.5 mm lateral to the midline, and 2.0–2.6 mm below the dorsal medullary surface). Arterial blood pressure was recorded by a strain gauge manometer. End-tidal CO_2_ partial pressure was measured by an infrared CO_2_ analyzer (Datex, CD-102; Normocap, Helsinki, Finland). Integrated phrenic and abdominal activities as well as the signals of the other variables studied were recorded on an eight-channel rectilinearly writing chart recorder (model 8K20; NEC San-ei, Tokyo, Japan). Cardiorespiratory variables were also acquired and analyzed using a personal computer, equipped with an analog-to-digital interface (Digidata 1200; Molecular Devices, Sunnyvale, CA) and appropriate software (Axoscope; Molecular Devices).

### Microinjection procedures

**Figure 1:**
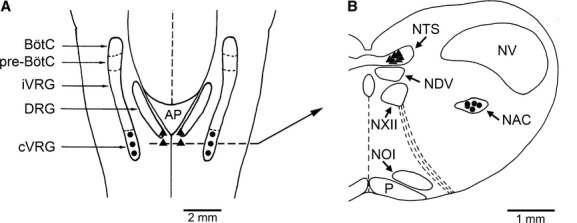
Localization of injection sites. (A) A diagrammatic representation of a dorsal view of the medulla oblongata of the rabbit showing where bilateral microinjections of antitussive drugs have been performed into the cNTS (▲) and the cVRG (•), respectively. Abbreviations: AP, area postrema; BötC, Bötzinger complex; cVRG, caudal ventral respiratory group; DRG, dorsal respiratory group; iVRG, inspiratory portion of the ventral respiratory group; preBötC, pre-Bötzinger complex. (B) diagram of a coronal section of the medulla oblongata at the levels indicated in panel A (dashed lines) showing the location of representative sites where the microinjections have been performed. NAC, nucleus ambiguous caudalis; NDV, nucleus dorsalis nervi vagi; NOI, nucleus olivaris inferior; NTS, nucleus tractus solitarii; NV, nucleus tractus spinalis nervi trigemini; NXII, nucleus nervi hypoglossi; P, tractus pyramidalis. The atlas of Meessen and Olszewski (1949) and the atlas of Shek et al. (1986) were used for comparison (see also Mutolo et al. 2007).

Microinjection procedures have been fully described in previous reports (Bongianni et al. 2005; Mutolo et al. 2007, 2008b, 2009, 2010, 2012; Cinelli et al. 2012). Bilateral microinjections were performed at two different sites along the rostrocaudal extent of the cNTS, and particularly into the lateral commissural NTS. They were approximately as follows: the first site at the level of the caudal-most end of the area postrema, 0.6–0.8 mm lateral to the midline and 0.7–0.8 mm below the dorsal medullary surface, and the second 0.5 mm more caudal, usually 0.4–0.5 mm lateral to the midline and 0.7–0.8 mm below the dorsal medullary surface. The stereotaxic coordinates were selected according to the atlas of Meessen and Olszewski (1949). Bilateral microinjections were also performed into the cVRG at sites defined by stereotaxic coordinates derived from prior extracellular recordings. Bilateral microinjections of the selected drug were made into the cVRG at three different sites, starting from ∼2.0 mm caudal to the obex, and continuing along the rostrocaudal extent of the VRG subregion at intervals of 0.5 mm. These procedures were followed to affect as much as possible the entire population of either cough-related second-order neurons of the cNTS (Kubin and Davies 1995; Kubin et al. 2006; Mutolo et al. 2007, 2009) or expiration-related neurons of the cVRG (Bongianni et al. 2005; Mutolo et al. 2009, 2010; Cinelli et al. 2012). In both cats and rats, the localization of central terminations of lung RARs and, therefore, of second-order neurons in this afferent pathway is the cNTS in its medial aspect and, especially, in the lateral portion of the commissural subnucleus of the NTS (Kubin and Davies 1995; Kubin et al. 2006). The localization of second-order RAR cells has been confirmed in the rabbit by our observations on the effects of local blockade of glutamate transmission (Mutolo et al. 2007, 2009). However, despite tracing and neuropharmacological experiments (Canning and Mori 2010) showing in the guinea pig that second-order neurons of cough-related afferents are localized not only in the commissural NTS but also in more rostral NTS regions, the general conclusion provided in this study is that the discrepancy can be attributed to the different airway portions investigated, that is, intrathoracic trachea and bronchi in the studies by Mutolo et al. (2007, 2009) or extrathoracic trachea in the study by Canning and Mori (2010). Microinjections (30–50 nL) were performed via a glass micropipette (tip diameter 10–25 μm) by applying pressure using an air-filled syringe connected to the micropipette by polyethylene tubing. The volume of the injectate was measured directly by monitoring the movement of the fluid meniscus in the pipette barrel with a dissecting microscope equipped with a fine reticule. The time taken to inject the solution ranged from 5 to 10 sec. A single micropipette was used to perform in succession the microinjections. The time taken to perform all the microinjections was 4–5 min for the cNTS and 6–8 min for the cVRG. The following drugs were used: clonidine hydrochloride (0.5 and 5.0 mmol/L; Tocris Cookson, Bristol, U.K.) and tizanidine hydrochloride (0.5 and 5.0 mmol/L; Sigma-Aldrich), *α*_2_-adrenergic receptor agonists; yohimbine hydrochloride (10 mmol/L; Sigma-Aldrich), an *α*_2_-adrenergic receptor antagonist. Only one of these drugs was tested in each preparation unless otherwise stated. Microinjections with similar drug concentrations have been performed in previous studies and have been shown to be selective in in vivo preparations (Gatti et al. 1988; Herman et al. 2008; Mansur et al. 2010; Deyama et al. 2011). All drugs were dissolved in 0.9% NaCl solution. The pH was adjusted to 7.4 with either 0.1 N NaOH or 0.1 N HCl. Control injections of equal volumes of the vehicle solution were also made. The localization of injection sites is diagrammatically illustrated in Figure 1. The recovery process could be followed for a maximum of ∼2 h. However, in most instances, the recovery was attained by microinjections of 10 mmol/L yohimbine at the responsive sites.

### Intravenous administration of drugs

Each *α*_2_-adrenergic receptor agonist was also administered intravenously at repeated doses of 20 μg/kg for clonidine and 50 μg/kg for tizanidine, with a time interval of at least 20 min between injections. Yohimbine was administered intravenously (300 μg/kg) to reverse clonidine- or tizanidine-induced effects on cough responses. All drugs were dissolved in 0.9% NaCl solution. Control intravenous injections of the vehicle solution were also made. Drug concentrations were selected in preliminary trials and were in the same range as those employed in previous studies (McCrimmon and Lalley 1982; Abbadie et al. 1994; Hirata et al. 1995; Li and Dampney 1995).

### Stimulation procedures

Cough was induced by both mechanical and chemical stimulation of the tracheobronchial tree. Mechanical stimulation was performed by means of a 0.5-mm-diameter nylon fiber with a smoothed tip inserted through a lateral port of the tracheal cannula until the tip was judged to be near the carina and main bronchi (for further details see Bongianni et al. 2005; Mutolo et al. 2007, 2008b, 2010; Cinelli et al. 2012). Back and forth movements of the fiber (∼3 cm) aimed at touching repeatedly (∼1 time every second) the carina or nearby airway walls were made over periods of 4–5 sec. An interval of ∼1 min was scheduled between cough stimulations. As a rule, three stimulation trials were performed in succession before drug administration. These stimulation trials were also accomplished ∼5 min after the completion of all the microinjections and repeated at appropriate intervals (at least 4–5 min). Sneezing was induced by using a 0.3-mm-diameter nylon fiber with a smoothed tip inserted into one nostril and pushed two times forward 1.5 cm into the nose. This mechanical stimulation was gentle and short lasting (∼3 sec) to avoid as much as possible traumatic effects. Before nasal stimulation, the nylon fiber was positioned into one nostril for an extent (starting point) proved in preliminary trials to be suitable for the generation of consistent reflex responses (Korpáš and Tomori 1979; Mutolo et al. 2009, 2012). The sneeze reflex (three stimulation trials) was elicited after mechanically induced cough before and after drug administration only when the cough responses were abolished by *α*_2_-adrenergic receptor agonists (see Results).

Chemical stimulation of the tracheobronchial tree was performed by means of citric acid inhalation (Mutolo et al. 2009, 2012; Cinelli et al. 2012). Citric acid (1 mol/L, Sigma-Aldrich) was freshly dissolved in 0.9% NaCl solution and nebulized (particle diameter 80% from 0.5 to 8 μm; nebulization rate 0.5 mL/min) via an ultrasonic nebulizer (Projet, Artsana, Grandate, CO, Italy). The opening of the tracheal cannula, through which the rabbits were spontaneously breathing, was exposed to a steady stream of the nebulized citric acid solution for ∼3 sec. This short period proved to be adequate to avoid as much as possible tachyphylaxis (Mutolo et al. 2009). The interval between chemical challenges was >10 min (usually ∼15 min) as similar cough reflexes could be reliably obtained at minimal intervals of 7 min (Mutolo et al. 2009, 2012; Cinelli et al. 2012). Chemical stimulation was always applied 2–3 min after the three trials of mechanically induced cough. As a rule, chemical stimulation was performed both before and ∼15 min after the completion of the injections and repeated at appropriate intervals to follow the time course of the recovery process for a maximum of ∼2 h. Much care was taken to perform all stimulation procedures ≥6 min after each supplemental dose of pentobarbital to avoid the possible immediate influences of the injected bolus on both the breathing pattern and reflex responses.

### Histology

At the end of each experiment, the brain was perfused via a carotid artery with 0.9% NaCl solution and subsequently with 10% formalin solution. After at least a 48-h immersion in 10% formalin solution, the brain was placed in a hypertonic sucrose solution. Frozen 20-μm coronal sections stained with cresyl violet were used for the histological control of pipette tracks and injection sites. The atlas of Meessen and Olszewski (1949) and the atlas of Shek et al. (1986) were used for comparison (see also Mutolo et al. 2007). Details on the localization of pipette tracks and injection sites have already been described in several previous reports (Bongianni et al. 2005; Mutolo et al. 2008b, 2009, 2010, 2012; Cinelli et al. 2012).

### Data collection and analysis

Respiratory variables were measured during eupneic breathing and reflex responses. The inspiratory (*T*_I_) and expiratory (*T*_E_) times, as well as the total duration of the respiratory or cough cycle (*T*_T_) were measured on recordings of raw phrenic nerve activity. The respiratory frequency was subsequently calculated (breaths/min). Peak amplitude (arbitrary units) of the phrenic nerve activity and abdominal EMG activity was measured on integrated traces. Normalization of the amplitudes of phrenic and abdominal activities was performed by expressing them as a fraction (or percentage) of the highest achievable amplitude observed in each animal. The highest peak values were consistently observed during coughing. Therefore, all amplitudes have been expressed in relative units (RU; see, e.g., Bolser et al. 1999; Mutolo et al. 2007, 2008b, 2009, 2010, 2012; Cinelli et al. 2012). Breathing pattern variables were measured for an average of five consecutive breaths prior to and following drug bilateral microinjections into either the cNTS or the cVRG as well as prior and following intravenous administration. Furthermore, systolic and diastolic blood pressures were measured at 2 sec intervals; mean arterial pressure was calculated as the diastolic pressure plus one third of the pulse pressure. Owing to the small variations in respiratory and cardiovascular variables within each measurement period, average values were taken as single measurement for the purpose of analysis.

The cough motor pattern in response to mechanical or chemical stimulation of the tracheobronchial tree is usually characterized by repeated coughs. Each cough consists of an augmented phrenic burst (preparatory inspiration) immediately followed by a burst of expiratory abdominal activity (Bongianni et al. 2005; Mutolo et al. 2007, 2008a,b, 2009, 2010, 2012; Cinelli et al. 2012). In agreement with our previous results, repeated coughs usually started during stimulation and continued shortly after stimulus cessation. Respiratory variables of coughs (cough-related variables) included cough-related *T*_T_, *T*_I_, and *T*_E_, peak phrenic amplitude (RU), peak abdominal activity (RU), and cough number, that is, the number of coughs following each stimulation. Cough-related variables were measured and averaged before and after drug administration at the time when the maximum response occurred (three trials for mechanical stimulation and a single trial for citric acid inhalation). The average values of cough-related variables were taken as single measurements for subsequent statistical analysis (Sigma Stat; SPSS, Chicago, IL). In some cases, the first obvious response following mechanical stimulation of the tracheobronchial tree was a small-amplitude expiratory effort without a preceding preparatory inspiration (e.g., Bongianni et al. 2005; Mutolo et al. 2007, 2010; Cinelli et al. 2012). This pattern could fit more appropriately the definition of expiration reflex that is typically evoked by mechanical stimulation of the vocal folds (Korpáš and Tomori 1979; Widdicombe and Fontana 2006), but that can be also produced by mechanical stimulation of the tracheobronchial tree (Widdicombe 1954). For further details on this topic see our previous reports (Mutolo et al. 2007, 2008b, 2009, 2010). However, in our study an expiration reflex only occurred as the first motor event in a cough epoch, and its appearance was limited to a few occasions. Therefore, these expiratory responses were not considered for data analysis.

Sneezing responses induced by mechanical stimulation consisted of an attack of 3–5 sneezes. Each sneeze consisted of a preparatory augmented inspiration, followed by an intense burst of expiratory activity (e.g., Korpáš and Tomori 1979; Mutolo et al. 2009, 2012). For simplicity, we considered only some sneeze-related variables, that is, the number of expiratory thrusts and peak abdominal activity. Sneeze-related variables were measured and averaged (three trials) before and after drug microinjections into either the cNTS or the cVRG as well as after intravenous administration of drugs. Also in this case, average values were taken as single measurements for the purpose of analysis. Average values of cough- and sneeze-related variables observed under control conditions and at the time when the maximum response to drug administration occurred were considered (see Results). In each preparation, microinjections of clonidine and tizanidine were performed usually at two concentrations. The microinjections at the higher concentration were performed after complete recovery from the effects induced by the preceding microinjections. Comparisons between cough-related variables recorded under control conditions and after administrations of tizanidine were performed by means of the one-way repeated-measures analysis of variance (ANOVA) followed by Student–Newman–Keuls tests. Drug-induced cardiorespiratory changes in the remaining experimental conditions were evaluated by Student's paired *t*-tests. All reported values are means ± SEM; *P* < 0.05 was taken as significant.

## Results

### Effects of microinjections of clonidine and tizanidine on the cough reflex

**Figure 2:**
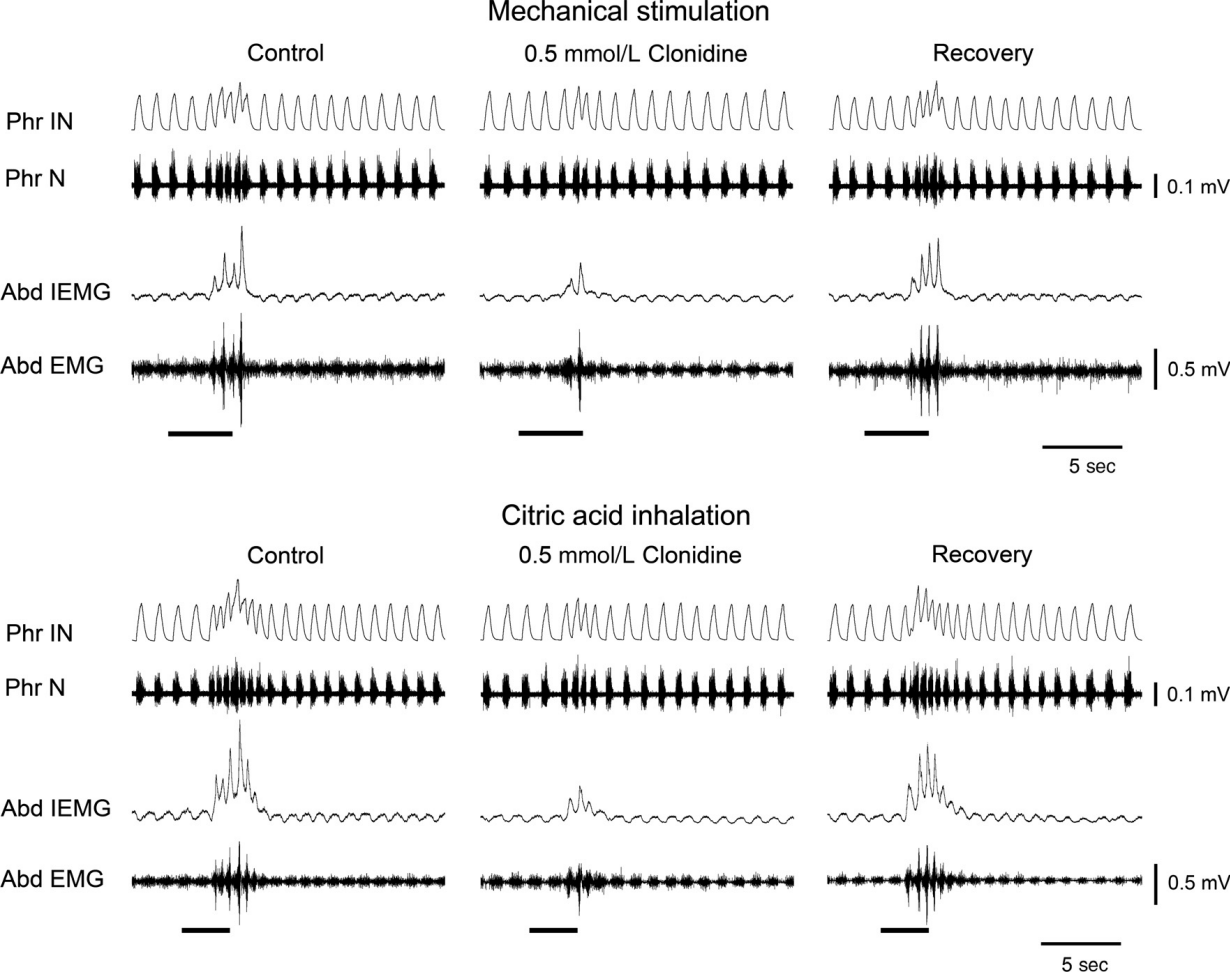
Effects of 0.5 mmol/L clonidine microinjected into the selected sites of the cNTS on cough reflex responses elicited by mechanical stimulation of the tracheobronchial tree and by the inhalation of 1 mol/L citric acid in one anesthetized spontaneously breathing rabbit. Reduction in cough responses 20 min after bilateral microinjections of clonidine, that is, when the maximum effect occurred. Recovery of cough responses was taken ∼90 min after injections. Stimulation periods marked by filled bars. Phr IN, phrenic integrated neurogram; Phr N, phrenic neurogram; Abd IEMG, abdominal integrated electromyographic activity; Abd EMG, abdominal electromyographic activity.

**Figure 3:**
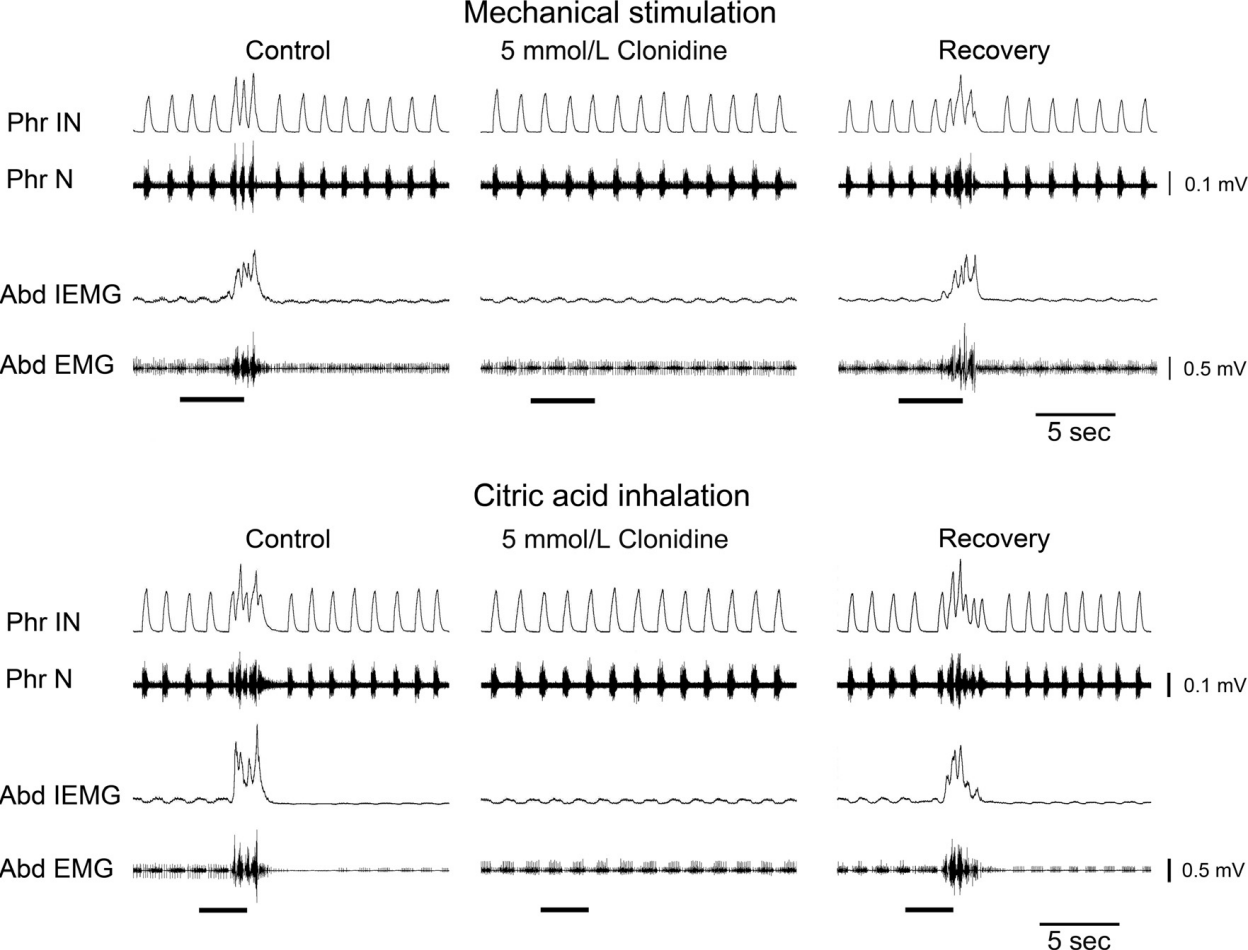
Depressant effects of 5 mmol/L clonidine microinjected into the selected sites of the cNTS on cough reflex responses elicited by mechanical stimulation of the tracheobronchial tree or by the inhalation of 1 mol/L citric acid in one anesthetized spontaneously breathing rabbit. Complete suppression of cough responses 20 min after bilateral microinjections of 5 mmol/L clonidine. Recovery of cough responses was taken ∼2 h after injections. Stimulation periods marked by filled bars. Phr IN, phrenic integrated neurogram; Phr N, phrenic neurogram; Abd IEMG, abdominal integrated electromyographic activity; Abd EMG, abdominal electromyographic activity.

Bilateral microinjections of 0.5 mmol/L clonidine (30–50 nL; 15–25 pmol) at the two selected cNTS sites were performed in six animals. Depressant effects were already present on mechanically induced cough 5 min after the microinjections while consistent and marked reductions in the cough response to both mechanical and chemical stimulation of the tracheobronchial tree were observed within ∼15 min (Fig. 2 and Table 1). The cough number and peak abdominal activity decreased, whereas the cough-related *T*_T_ increased due to a rise in both *T*_I_ and *T*_E_. Bilateral microinjections of 5 mmol/L clonidine (30–50 nL; 150–250 pmol) at the same medullary sites induced rapid (within 5 min) depression of mechanically induced cough. Cough responses to both types of stimulation were progressively depressed up to the complete suppression of them within ∼20 min (Fig. 3 and Table 1).

**Table 1:** Cough-related variables before and 15 min after bilateral microinjections of clonidine into the cNTS (*n* = 6)

	CN	*T*_T_ (sec)	*T*_I_ (sec)	*T*_E_ (sec)	PPA (RU)	PAA (RU)
Mechanical stimulation						
Control	3.3 ± 0.22	0.57 ± 0.02	0.38 ± 0.02	0.19 ± 0.01	0.60 ± 0.04	0.62 ± 0.03
0.5 mmol/L clonidine	1.7 ± 0.21**	0.74 ± 0.04*	0.47 ± 0.03*	0.27 ± 0.03*	0.55 ± 0.04	0.43 ± 0.04*
5 mmol/L clonidine	−	−	−	−	−	−
Citric acid inhalation						
Control	5.0 ± 0.52	0.50 ± 0.02	0.34 ± 0.02	0.16 ± 0.01	0.59 ± 0.02	0.60 ± 0.01
0.5 mmol/L clonidine	2.0 ± 0.26**	0.62 ± 0.02*	0.42 ± 0.02*	0.20 ± 0.01*	0.54 ± 0.04	0.38 ± 0.02**
5 mmol/L clonidine	−	−	−	−	−	−

Values are means ± SEM; *n*, number of animals; CN, cough number; *T*_T_, cycle duration; *T*_I_, inspiratory time; *T*_E_, expiratory time; PPA, peak phrenic activity in relative units (RU); PAA, peak abdominal activity in relative units (RU). Negative marks indicate the absence of cough responses. *P < 0.05; **P < 0.005 compared with control cough.

In other six preparations, bilateral microinjections of 0.5 mmol/L clonidine (30–50 nL; 15–25 pmol) at the selected sites of the cVRG produced depressant effects of mechanically induced cough within 5 min, whereas pronounced decreases in cough responses evoked by mechanical and chemical stimulation of the tracheobronchial tree were achieved within ∼15 min (Table 2). Cough responses induced by mechanical stimulation showed reductions in cough number and peak abdominal activity associated with increases in the cough-related *T*_T_ due to a rise in *T*_E_. Similar changes were observed in chemically induced cough responses, although cough-related *T*_T_ increased owing to significant changes in both *T*_I_ and *T*_E_. Clonidine at 5 mmol/L (30–50 nL; 150–250 pmol) microinjected into the cVRG always abolished cough responses within ∼20 min (Table 2). Cough suppressant effects developed progressively. They were already seen on mechanically induced cough within 5 min after the microinjections. As these cough responses displayed features similar to those already shown in Figures 2 and 3 for clonidine microinjections into the cNTS, cough-related variables before and after treatment have not been illustrated by original recordings, but only reported in Table 2.

**Table 2:** Cough-related variables before and 15 min after bilateral microinjections of clonidine into the cVRG (*n* = 6)

	CN	*T*_T_ (sec)	*T*_I_, (sec)	*T*_E_ (sec)	PPA (RU)	PAA (RU)
Mechanical stimulation						
Control	3.5 ± 0.23	0.62 ± 0.01	0.37 ± 0.01	0.24 ± 0.01	0.62 ± 0.02	0.59 ± 0.02
0.5 mmol/L clonidine	2.0 ± 0.25**	0.74 ± 0.03*	0.39 ± 0.01	0.35 ± 0.03*	0.57 ± 0.03	0.41 ± 0.03*
5 mmol/L clonidine	−	−	−	−	−	−
Citric acid inhalation						
Control	4.7 ± 0.42	0.51 ± 0.01	0.35 ± 0.01	0.16 ± 0.01	0.57 ± 0.02	0.58 ± 0.01
0.5 mmol/L clonidine	2.5 ± 0.22**	0.71 ± 0.04*	0.43 ± 0.01*	0.27 ± 0.03*	0.51 ± 0.03	0.32 ± 0.05**
5 mmol/L clonidine	−	−	−	−	−	−

Values are means ± SEM; *n*, number of animals; CN, cough number; *T*_T_, cycle duration; *T*_I_, inspiratory time; *T*_E_, expiratory time; PPA, peak phrenic activity in relative units (RU); PAA, peak abdominal activity in relative units (RU). Negative marks indicate the absence of cough responses. *P < 0.05; **P < 0.005 compared with control cough.

Bilateral microinjections (*n* = 5) of 0.5 mmol/L tizanidine (30–50 nL; 15–25 pmol) into the cNTS progressively depressed cough responses up to the complete suppression of them within ∼20 min. By contrast, bilateral microinjections (*n* = 5) of 0.5 mmol/L tizanidine (30–50 nL; 15–25 pmol) into the cVRG did not cause changes in the cough reflex, whereas tizanidine at 5 mmol/L (same preparations) produced consistent reductions in cough responses induced by both mechanical and chemical stimulation of the tracheobronchial tree (Table 3). The cough number and peak abdominal activity decreased, whereas cough-related *T*_T_ increased due to rise in *T*_E_. The onset of depressant effects (mechanically induced cough) was similar to that above reported for clonidine.

**Table 3:** Cough-related variables before and 15 min after bilateral microinjections of tizanidine into the cVRG (*n* = 5)

	CN	*T*_T_ (sec)	*T*_I_ (sec)	*T*_E_ (sec)	PPA (RU)	PAA (RU)
Mechanical stimulation						
Control	3.8 ± 0.20	0.55 ± 0.03	0.37 ± 0.03	0.18 ± 0.01	0.53 ± 0.04	0.67 ± 0.03
0.5 mmol/L tizanidine	3.6 ± 0.19	0.56 ± 0.03	0.37 ± 0.03	0.19 ± 0.02	0.52 ± 0.04	0.65 ± 0.04
5 mmol/L tizanidine	2.6 ± 0.40*	0.71 ± 0.07*	0.37 ± 0.03	0.34 ± 0.05*	0.52 ± 0.05	0.46 ± 0.08*
Citric acid inhalation						
Control	5.8 ± 0.21	0.54 ± 0.03	0.38 ± 0.02	0.17 ± 0.01	0.55 ± 0.06	0.69 ± 0.02
0.5 mmol/L tizanidine	5.2 ± 0.37	0.56 ± 0.04	0.39 ± 0.02	0.18 ± 0.02	0.58 ± 0.06	0.62 ± 0.06
5 mmol/L tizanidine	4.0 ± 0.44*	0.74 ± 0.08*	0.41 ± 0.03	0.33 ± 0.07*	0.59 ± 0.09	0.38 ± 0.06*

Values are means ± SEM; *n*, number of animals; CN, cough number; *T*_T_, cycle duration; *T*_I_, inspiratory time; *T*_E_, expiratory time; PPA, peak phrenic activity in relative units (RU); PAA, peak abdominal activity in relative units (RU).

* *P* < 0.05 compared with control cough and 0.5 mmol/L tizanidine.

In all cases, when the cough reflex was completely abolished by drug application, mechanical stimulation of the tracheobronchial tree at higher frequency and/or duration as well as citric acid inhalation for longer periods failed to evoke any cough response. With the lower concentrations of clonidine, a complete recovery of the cough reflex was seen after ∼90 min from the completion of the microinjections performed into either the cNTS or the cVRG, whereas with the higher concentrations the time course of recovery was slower. In fact, a complete, spontaneous recovery was observed after ∼2 h in some preparations (*n* = 2 for the cNTS and *n* = 2 for the cVRG). In other preparations (*n* = 4 for the cNTS and *n* = 4 for the cVRG), clonidine-induced depressant effects on the cough reflex were completely reverted by microinjections of 10 mmol/L yohimbine (30–50 nL, 300–500 pmol) at the responsive sites. A complete recovery of cough responses was observed within ∼60 min following microinjections of 5 mmol/L tizanidine into the cVRG. We did not wait for the spontaneous recovery following tizanidine microinjections into the cNTS, but depressant effects on the cough reflex were always reverted by microinjections of 10 mmol/L yohimbine at the responsive sites.

### Effects of microinjections of clonidine and tizanidine on the sneeze reflex

**Figure 4:**
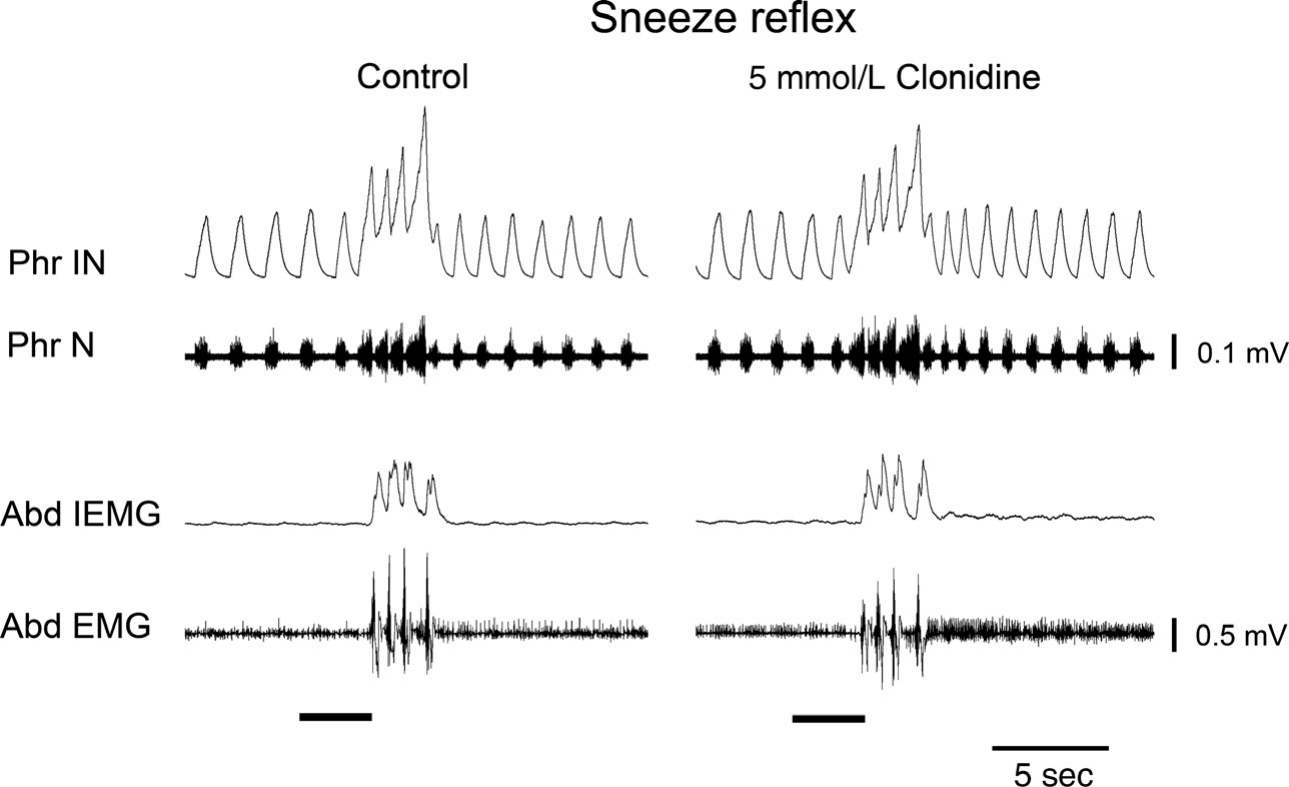
Persistence of the sneeze reflex after bilateral microinjections of 5 mmol/L clonidine into the cNTS in one anesthetized spontaneously breathing rabbit. The sneeze reflex was induced by mechanical stimulation of the nasal mucosa (filled bars) before (control) and ∼20 min after the completion of the bilateral microinjections, that is, when the cough reflex was abolished. Phr IN, phrenic integrated neurogram; Phr N, phrenic neurogram; Abd IEMG, abdominal integrated electromyographic activity; Abd EMG, abdominal electromyographic activity.

Noticeably, when the cough reflex was suppressed by drug application, mechanical stimulation of the nasal mucosa still produced the sneeze reflex. Clonidine at 5 mmol/L microinjected into either the cNTS or the cVRG induced no significant changes in the number of expiratory thrusts (from 3.5 ± 0.21 to 3.4 ± 0.19 for cNTS and from 3.5 ± 0.23 to 3.3 ± 0.22 for cVRG) and in peak abdominal activity (from 0.47 ± 0.03 to 0.50 ± 0.04 RU for cNTS and from 0.46 ± 0.03 to 0.52 ± 0.07 RU for cVRG). Similarly, no significant changes in the number of expiratory thrusts (from 3.3 ± 0.25 to 3.6 ± 0.22) and in peak abdominal activity (from 0.45 ± 0.02 to 0.50 ± 0.03 RU) were observed in response to microinjections of 0.5 mmol/L tizanidine into the cNTS. A typical example of the persistence of sneeze reflex responses for microinjections of 5 mmol/L clonidine into the cNTS is reported in Figure 4.

### Effects of microinjections of clonidine and tizanidine on cardiorespiratory variables during eupneic breathing

For simplicity, only a table containing mean values of cardiorespiratory variables during eupneic control breathing in all studied animals has been presented (Table 4). Although our attention was mainly focused on cough responses, we also observed that bilateral microinjections of clonidine and tizanidine into the cNTS and the cVRG did not affect respiratory variables during eupneic control breathing that at the time when the maximum cough reflex responses occurred were quite similar to those presented in Table 4 (see also control recordings before reflex responses in Figs 2, 3, and 4). Similarly, none of the employed drugs significantly affected mean arterial blood pressure that was always between 97 and 101 mmHg at the time when the maximum cough reflex responses occurred (98.4 ± 0.3 and 97.9 ± 0.5 mmHg following clonidine and tizanidine, respectively; data from all trials, see for comparison Table 4). For a general evaluation of cardiorespiratory variables in the rabbit under control conditions, see also our previous reports (Bongianni et al. 2005; Mutolo et al. 2007, 2008b, 2009, 2010).

**Table 4:** Cardiorespiratory variables during eupneic breathing in all studied animals (*n* = 37)

*T*_T_ (sec)	1.22 ± 0.02
*T*_I_ (sec)	0.48 ± 0.004
*T*_E_ (sec)	0.74 ± 0.02
PPA (RU)	0.57 ± 0.01
PAA (RU)	0.05 ± 0.001
MAP (mmHg)	98.7 ± 0.2

Values are means ± SEM; *n*, number of animals; *T*_T_, cycle duration; *T*_I_, inspiratory time; *T*_E_, expiratory time; PPA, peak phrenic activity in relative units (RU); PAA, peak abdominal activity in relative units (RU); MAP, mean arterial pressure.

### Effects of intravenous administration of clonidine and tizanidine on the cough reflex

**Figure 5:**
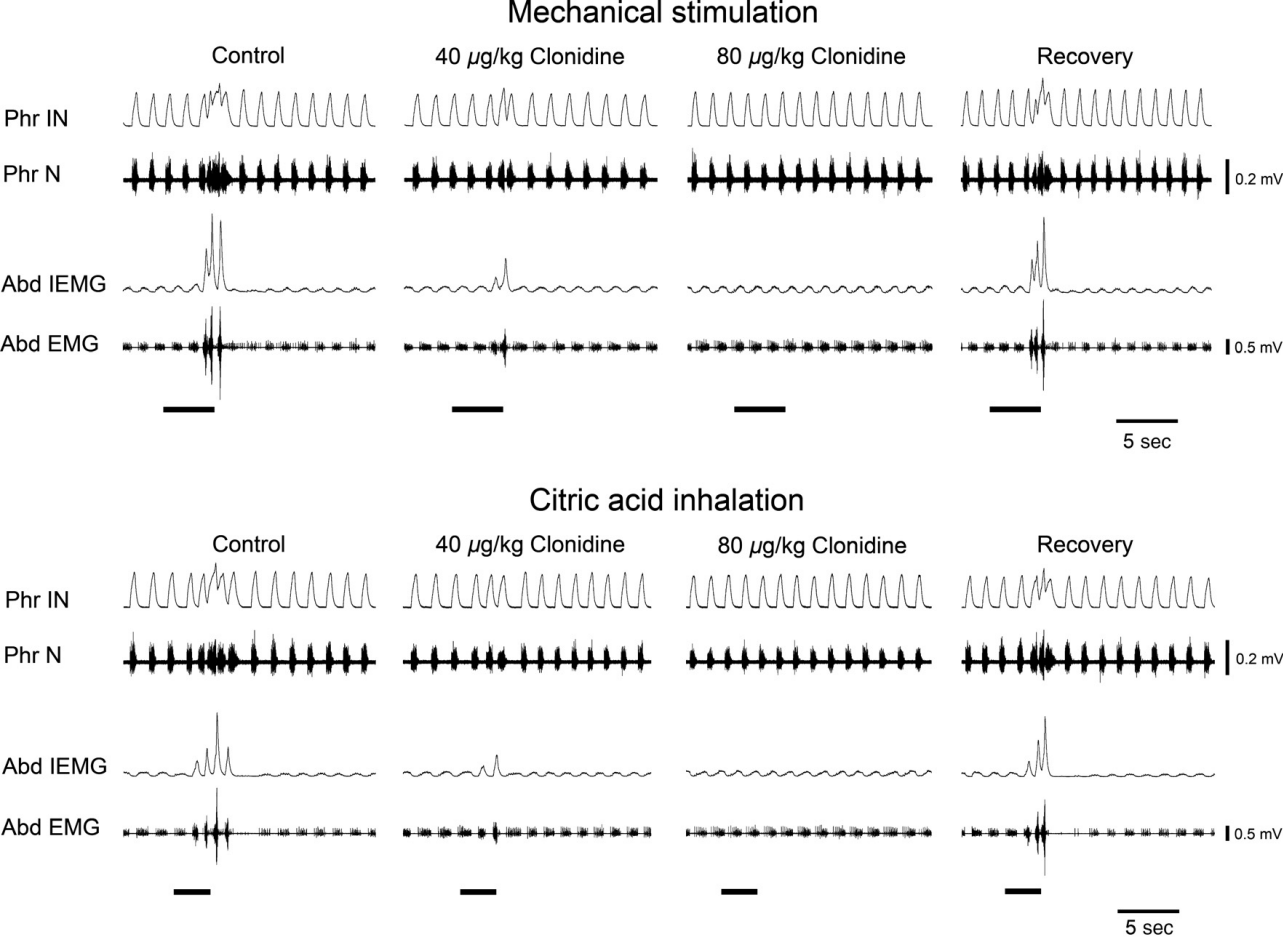
Changes in the cough reflex induced by mechanical stimulation of the tracheobronchial tree or by the inhalation of 1 mol/L citric acid induced by intravenous administration of clonidine in one anesthetized spontaneously breathing rabbit. Reduction and suppression of the cough reflex ∼20 min after the administration of 40 μg/kg and 80 μg/kg clonidine, respectively. In both cases, the complete recovery of cough reflex was obtained ∼15 min after the intravenous administration of yohimbine (300 μg/kg). Stimulation periods marked by filled bars. Phr IN, phrenic integrated neurogram; Phr N, phrenic neurogram; Abd IEMG, abdominal integrated electromyographic activity; Abd EMG, abdominal electromyographic activity.

Clonidine was administered intravenously (*n* = 6) at repeated doses of 20 μg/kg, with a time interval of at least 20 min in between. As shown in Table 5 and Figure 5, cumulative doses of 40–60 μg/kg always strongly reduced cough reflex responses, whereas cumulative doses of 80–120 μg/kg abolished the cough reflex. The observed effects developed within 20 min after the last injection and were completely reverted by the intravenous administration of yohimbine (300 μg/kg). The recovery was rapid and complete within 15 min from yohimbine administration. Similarly (Table 5), intravenous administration of tizanidine (*n* = 4) completely abolished both mechanically and chemically induced cough responses. Tizanidine was administered at repeated doses of 50 μg/kg (time interval of at least 20 min). Cumulative doses of 150 μg/kg in all cases strongly reduced cough reflex responses, whereas cumulative doses of 300 μg/kg abolished cough reflex responses within ∼20 min after the last injection. Tizanidine-induced effects were completely reverted within ∼15 min after the intravenous administration of yohimbine (300 μg/kg). When the cough reflex was abolished by drug application, mechanical stimulation of the tracheobronchial tree at higher frequency or duration (Mutolo et al. 2010) as well as citric acid inhalation for a longer period failed to evoke any cough response. As already shown following clonidine microinjections (Fig. 4), the sneeze reflex produced by the mechanical stimulation of the nasal mucosa did not display appreciable changes (not shown).

**Table 5:** Cough-related variables before and after intravenous administration of clonidine (*n* = 6) and tizanidine (*n* = 4)

	CN	*T*_T_ (sec)	*T*_I_ (sec)	*T*_E_ (sec)	PPA (RU)	PAA (RU)
Mechanical stimulation						
Control	3.6 ± 0.21	0.58 ± 0.02	0.39 ± 0.02	0.19 ± 0.01	0.58 ± 0.03	0.63 ± 0.03
40–60 μg/kg clonidine	1.5 ± 0.22**	0.84 ± 0.04*	0.53 ± 0.04*	0.31 ± 0.04*	0.52 ± 0.02	0.43 ± 0.04*
80–120 μg/kg clonidine	−	−	−	−	−	−
Citric acid inhalation						
Control	4.7 ± 0.42	0.51 ± 0.01	0.35 ± 0.01	0.16 ± 0.01	0.57 ± 0.02	0.58 ± 0.01
40–60 μg/kg clonidine	1.7 ± 0.21**	0.72 ± 0.05*	0.46 ± 0.03*	0.26 ± 0.02*	0.53 ± 0.03	0.36 ± 0.02**
80–120 μg/kg clonidine	−	−	−	−	−	−
Mechanical stimulation						
Control	3.5 ± 0.29	0.57 ± 0.02	0.38 ± 0.03	0.19 ± 0.01	0.61 ± 0.03	0.66 ± 0.04
150 μg/kg tizanidine	1.5 ± 0.29*	0.82 ± 0.02*	0.49 ± 0.03*	0.33 ± 0.03*	0.51 ± 0.03	0.42 ± 0.06*
300 μg/kg tizanidine	−	−	−	−	−	−
Citric acid inhalation						
Control	4.2 ± 0.42	0.51 ± 0.01	0.35 ± 0.01	0.16 ± 0.01	0.57 ± 0.02	0.58 ± 0.01
150 μg/kg tizanidine	1.3 ± 0.22**	0.70 ± 0.03*	0.44 ± 0.02*	0.27 ± 0.02*	0.55 ± 0.03	0.40 ± 0.02**
300 μg/kg tizanidine	−	−	−	−	−	−

Values are means ± SEM; *n*, number of animals; CN, cough number; *T*_T_, cycle duration; *T*_I_, inspiratory time; *T*_E_, expiratory time; PPA, peak phrenic activity in relative units (RU); PAA, peak abdominal activity in relative units (RU). Negative marks indicate the absence of cough responses. *P < 0.05; **P < 0.005 compared with control cough.

Intravenous administration of both *α*_2_-adrenergic receptor agonists did not affect respiratory variables during eupneic control breathing that at the time when the maximum cough reflex responses occurred were similar to those reported in Table 4 (see also control recordings before clonidine-induced reflex responses in Fig. 5). Each dose of clonidine caused, immediately after each injection, increases in mean arterial blood pressure from 99.2 ± 0.6 to 117.0 ± 2.5 mmHg (range of changes ∼10–25 mmHg) due to the peripheral action on *α*_2_-adrenergic receptors (e.g., Hirata et al. 1995; Philipp et al. 2002; Kaczynska and Szereda-Przestaszewska 2006). The maximum cardiovascular effects were achieved within ∼30 sec and a complete recovery of control values in mean arterial blood pressure (98.8 ± 1.0 mmHg) was observed within ∼3 min. Thereafter, mean arterial blood pressure progressively decreased to 85.3 ± 1.7 mmHg, that is, ∼10–20 mmHg below control levels at the time when the cough reflex was strongly reduced or completely abolished. The effects of the intravenous administration of tizanidine on blood pressure were quite similar to those obtained with clonidine. Therefore, they have been not reported to simplify the presentation. Also changes in arterial blood pressure induced by both *α*_2_-adrenergic receptor agonists were completely reverted by the intravenous administration of yohimbine.

### Controls

In five additional preparations, bilateral microinjections of 5 mmol/L clonidine or 5 mmol/L tizanidine performed at medullary locations sufficiently far from the responsive sites (e.g., Nicholson 1985; Mutolo et al. 2007, 2008b, 2009, 2012) did not cause appreciable changes in the pattern of breathing and cough reflex responses. They were made (for comparisons see the atlas of Meessen and Olszewski 1949) into the nucleus cuneatus (3 trials) or the nucleus tractus spinalis nervi trigemini (4 trials) as well as >0.8 mm caudal to the responsive sites of the cNTS (3 trials) or the cVRG (2 trials). Control injections of equal volumes of the vehicle solution at the responsive sites were ineffective (3 trials in different preparations for each investigated medullary region). The localization of the injection sites (Fig. 1) was confirmed by histological control. Examples of typical placements of the micropipette tips within the cNTS or the cVRG have already been illustrated in previous reports (Bongianni et al. 2005; Mutolo et al. 2007, 2010; Cinelli et al. 2012). Control inhalations of nebulized physiological saline did not produce any change in respiratory activity. Control intravenous administration of the vehicle solution did not cause any appreciable cardiorespiratory effects.

## Discussion

This is the first study that provides evidence that activation of *α*_2_-adrenergic receptors by microinjections of clonidine and tizanidine into the cNTS or the cVRG has suppressant effects on cough responses induced by mechanical stimulation of the tracheobronchial tree or by citric acid inhalation in the anesthetized rabbit. Downregulation of the cough reflex was also observed after intravenous administration of both drugs. Taken together, the results suggest that medullary *α*_2_-adrenergic receptors exert an inhibitory effect in the central sensory pathways involved in the genesis of the cough motor pattern. Present results confirm that both the cNTS and the cVRG are important components of the neural system involved in the central regulation of cough (e.g., Bongianni et al. 2005; Mazzone et al. 2005; Mutolo et al. 2007, 2008b, 2009, 2010, 2012; Poliacek et al. 2007, 2010; Canning and Mori 2010, 2011; Cinelli et al. 2012). Furthermore, these medullary regions as well as the activation of *α*_2_-adrenergic receptors appear to be specifically involved in the mediation of the cough reflex as the sneeze reflex was not affected by local and systemic administration of clonidine and tizanidine.

### Methodological considerations and general comments

We have already provided details about the microinjection techniques used, as well as a discussion on their reliability and the spread of the injectate (e.g., Bongianni et al. 2005; Mutolo et al. 2007, 2008b, 2009, 2010, 2012; Cinelli et al. 2012). Injection sites were selected by using stereotaxic coordinates as well as extracellular recordings from expiratory neurons of the cVRG (for details see Bongianni et al. 2005; Mutolo et al. 2010; Cinelli et al. 2012; see Von Euler 1986 for a review). Our previous observations on the spread of the injectate ≤50 nL (Mutolo et al. 2005) are in agreement with theoretical calculations by Nicholson (1985), suggesting that a volume of 50 nL should spread <385 μm in any direction from the injection site. Accordingly, microinjections of clonidine and tizanidine into regions sufficiently away from the responsive sites did not affect the cough reflex. The specificity of drug-induced effects is also supported by the absence of changes in cough reflex responses following control bilateral microinjections of the vehicle solution. Evidence on the specificity of *α*_2_-adrenergic receptor activation by clonidine and tizanidine is also provided by the antagonistic effects displayed by the *α*_2_-adrenergic receptor antagonist yohimbine both during microinjections and intravenous administration of drugs.

In this study, no consistent effects on the eupneic pattern of breathing were observed in response to clonidine or tizanidine. Varied results have been obtained with intravenous administration of clonidine in different animal species; that is, either increases or decreases in respiratory frequency in unanesthetized (Hedrick et al. 1994) and anesthetized (McCrimmon and Lalley 1982; Hedrick et al. 1994; Kaczynska and Szereda-Przestaszewska 2006) animals, respectively. This suggests a possible role of anesthesia and state of vigilance in clonidine-induced effects on the breathing pattern. In humans, the absence of breathing depressant effects following oral or intravenous administration of clonidine has been reported in healthy volunteers (Bailey et al. 1991; Ooi et al. 1991) and sleep apnea patients following surgery (Pawlik et al. 2005). However, a possible controversy between no effects versus limited effects (Jarvis et al. 1992) on human breathing following the administration of *α*_2_-adrenergic receptor agonists may result from drug-induced changes in the state of vigilance (e.g., Voituron et al. 2012 also for further references). As clonidine easily penetrates the blood–brain barrier (Panagiotidis et al. 1993), its ability to alter the respiratory activity may imply a central origin of the response. Accordingly, central application of *α*_2_-adrenergic receptor agonists has been reported to inhibit respiration in experimental animals (Burton et al. 1990; see Kaczynska and Szereda-Przestaszewska 2006 for further references). The absence of respiratory changes in our preparations may be related to the animal species employed as well as to the level and type of anesthesia.

Bilateral microinjections of clonidine and tizanidine into the cNTS and the cVRG did not affect mean arterial blood pressure. This finding is not surprising as there is general agreement that the rostral ventrolateral medulla, the final relay station of the baroreceptor reflex pathway in the brain, is the primary site of action of clonidine-like drugs (Szabo 2002 also for further references). However, decreases in mean arterial blood pressure after microinjections of clonidine into the commissural NTS have been recently described in rats (Bhuiyan et al. 2009). This discrepancy may be related to differences in the type of preparation, the microinjection technique, and the concentrations of clonidine employed. As to the cardiovascular effects of the intravenous administration of *α*_2_-adrenergic receptor agonists, our results are consistent with previous findings (e.g., Hirata et al. 1995; Philipp et al. 2002; Kaczynska and Szereda-Przestaszewska 2006). Intravenous administration of clonidine and tizanidine evoked a biphasic blood pressure response: a short-lived rise followed by prolonged hypothension. This is a typical cardiovascular response to intravenous administration of *α*_2_-adrenergic receptor agonists also in humans and other species. The pressor component of the blood pressure effects depends on the activation of *α*_2_-adrenergic receptors on the vascular smooth muscle cells, whereas hypothension is due to their excitation within the rostral ventrolateral medulla. In this context, it can be mentioned that Poliacek et al. (2011) reported that blood pressure changes could alter tracheobronchial cough in cats. Nevertheless, it should be recalled that clonidine induced decreases in arterial blood pressure, that is, changes that are expected to increase and not to decrease the cough reflex response. In addition, downregulation of the cough reflex was also observed in response to clonidine microinjected into the cNTS and the cVRG in the absence of any change in arterial blood pressure.

### Effects of clonidine and tizanidine on the cough reflex

Our results show that the activation of *α*_2_-adrenergic receptors is involved in the regulation of the cough reflex at the level of both the cNTS and the cVRG. During mechanical and chemical stimulation of the tracheobronchial tree, changes in cough-related variables induced by the lower concentration of clonidine within the cNTS (Table 1) and the cVRG (Table 2) recall those previously observed during mechanically induced coughing after microinjections of low concentrations of [D-Ala^2^,N-Me-Phe^4^,Gly^5^-ol] enkephalin(DAMGO), a *μ*-opioid agonist, and baclofen, a GABA_B_ receptor agonist, into the same medullary regions (Mutolo et al. 2008b, 2010). Interestingly, peak phrenic activity did not change. The differential effects observed on the inspiratory and expiratory motor output support the view of a different regulation of the inspiratory and expiratory components of the cough motor pattern (Bolser and Davenport 2002; Mutolo et al. 2008b, 2010). However, as in our previous reports (Mutolo et al. 2008b, 2010), clonidine also increased cough-related *T*_T_ because of increases in both cough-related *T*_I_ and *T*_E_. As peak phrenic amplitude did not significantly changed, the increases in *T*_I_ imply a reduced rate of rise of inspiratory activity (inspiratory drive) and, therefore, inspiratory depressant effects (Von Euler 1986). Increases in cough-related *T*_E_ similar to those reported in this study were observed not only after DAMGO or baclofen microinjections into the cNTS and the cVRG but also after *N*-methyl-D-aspartate receptor blockades within the cNTS (Mutolo et al. 2007, 2009). Noticeably, present and previous results from our laboratory (Mutolo et al. 2008b, 2010; Cinelli et al. 2012) agree, at least in part, with the assumption by Bolser and colleagues (Bolser et al. 1999, 2006) on the presence of a cough-gating mechanism mainly derived from studies on the differential effects of antitussive drugs on the cough and breathing pattern. The present results are in general agreement with those previously described by Bolser et al. (1999) for different antitussive drugs or those obtained by making use of codeine microinjections into the cVRG of the cat (Poliacek et al. 2010). However, as in our previous reports (Mutolo et al. 2007, 2008b, 2009, 2010, 2012; Cinelli et al. 2012), we have found not only changes in the cough number but also in the time components of the cough reflex. Although the reasons of these discrepancies are obscure, in agreement with our previous interpretation, we have tentatively attributed them to differences in the animal species, experimental conditions, and characteristics of the employed drugs. Furthermore, the rostrocaudal extent of the neuronal population affected by the injectate of antitussive drugs is much greater in our experiments. As already suggested for the antitussive effects of DAMGO and baclofen at the level of the cNTS (Mutolo et al. 2008b), we hypothesize that clonidine- and tizanidine-induced effects on the cough reflex may be due, at least in part, to reductions in glutamate release by presynaptic inhibition operated through *α*_2_-adrenergic receptors at the level of the central terminals of cough-related vagal afferents. In agreement with this hypothesis, it has been reported that clonidine can inhibit synaptic glutamate release from primary afferent nerves to spinal dorsal horn neurons, a mechanism of action that has been suggested to underlie analgesia produced by *α*_2_-adrenergic receptor agonists at the level of the spinal cord (Ueda et al. 1995; Pan et al. 2008). In this context it can be also recalled that *α*_2_-adrenergic receptor agonists may have other functions in the nervous system. They not only inhibit the release of norepinephrine by means of presynaptic receptors, but can also regulate the exocytosis of a number of other neurotransmitters (e.g., Ono et al. 1991; Philipp et al. 2002). More interestingly, *α*_2_-adrenergic receptors may be also located postsynaptically and their activation could lead to inhibition of postsynaptic neurons (e.g., Kawamata et al. 1997; Philipp et al. 2002 also for further references). Thus, postsynaptic inhibition of cNTS second-order neurons of the cough afferent pathway could also be suggested to play a role in the *α*_2_-adrenergic receptor-mediated cough suppressant effects.

Similar synaptic mechanisms can be suggested for the antitussive action of *α*_2_-adrenergic receptor agonists microinjected into the cVRG, that is, both pre- and postsynaptic actions of the employed drugs. As microinjections in the cVRG affected both the expiratory and inspiratory components of the cough reflex, we have to admit, in agreement with our previous suggestions (Bongianni et al. 2005; Mutolo et al. 2009, 2010; Cinelli et al. 2012), that, instead of expiratory cVRG neurons, more conceivably other types of cVRG neurons are involved. These latter neurons, either quiescent or with different respiratory or nonrespiratory discharge patterns, possibly receive cough-related inputs and project to brainstem respiration-related regions including the expiratory neuronal population of the cVRG (for references see Bongianni et al. 2005; Mutolo et al. 2009, 2010; Cinelli et al. 2012). Although it has been proved that expiratory cVRG neurons receive indirect inputs from SARs and RARs (e.g., Kubin and Davies 1995; Iscoe 1998; Sant'Ambrogio and Widdicombe 2001; Kubin et al. 2006), we believe that similar afferent inputs could conceivably also impinge on other types of cVRG neurons. This interpretation is also consistent with the absence of changes in expiratory abdominal activity following microinjections of *α*_2_-adrenergic receptor agonists.

The potential sources of adrenergic inputs to the cNTS and the cVRG may involve several sites in the central nervous system (see, e.g., Schreihofer and Guyenet 1997; Bhuiyan et al. 2009; Sevigny et al. 2012 also for further references), including the NTS (A2 cell group), ventrolateral (A1/C1 cell groups) and dorsomedial medulla (C3 cell group), and pons (A5 cell group). Adrenergic pathways have been identified in the NTS (Feldman and Moises 1988; Hayward et al. 2002), nucleus ambiguous, and ventral respiratory group (Ellenberger et al. 1990). However, the conditions under which *α*_2_-adrenergic receptors in these regions are brought into action to affect the cough reflex are at present only matter of speculation.

Surprisingly, clonidine and tizanidine displayed differences in the antitussive effect according to the brainstem structure in which they were microinjected. This could be tentatively explained by differences in pharmacological properties of the two drugs. It is well known that clonidine shows higher affinity than tizanidine to *α*_2_-adrenergic receptors (Muramatsu and Kigoshi 1992). Although tizanidine displays higher affinity than clonidine to imidazoline receptors (Muramatsu and Kigoshi 1992; Szabo 2002), the antitussive action of both drugs seems to be primarily, if not exclusively, mediated by *α*_2_-adrenergic receptors as it was reverted by the *α*_2_-adrenergic receptor antagonist yohimbine.

Actually, the effects of *α*_2_-adrenergic receptor agonist microinjections displayed a relatively rapid onset, but they developed progressively and reached a maximum with a fairly long latency. This time course is similar to that of other antitussive drugs such as DAMGO, baclofen, and CP-99,994 (Mutolo et al. 2008b, 2010), and may be related to their action on metabotropic receptors that require longer activation time than ionotropic receptors. In addition, as all these drugs probably act mainly at the presynaptic level to affect the release of other neurotransmitters, this implies another time delay. However, the mechanism underlying this long-lasting latency should be complex, and a satisfying interpretation is not at present available. The latency of response may also depend on the spread of the injectate to a sufficient number of responsive neurons surrounding the pipette tip.

Clonidine and tizanidine intravenously administered induced the complete suppression of cough responses displaying, however, different effective antitussive potency. This could be related to the fact that both drugs penetrate the blood–brain barrier (see, e.g., Panagiotidis et al. 1993; Hirata et al. 1995 also for further references), but clonidine has a higher affinity to the *α*_2_-adrenergic receptors by about 3 times and a higher lipophilicity and larger pKa than tizanidine (Takayanagi et al. 1985; Kawamata et al. 1997). It should also be kept in mind that the effects of systemic administration of both drugs are fairly complex owing to their probable simultaneous action on different neural structures. This makes it difficult for a detailed interpretation of the individual changes in cough-related variables. Although clonidine and tizanidine have an action at central level, we cannot exclude that part of the antitussive effects as systemic administration of these drugs could have also occurred via peripheral *α*_2_-adrenergic receptors, such as those located on sensory nerves in the airways of the guinea pigs (see also O'Connell et al. 1994). Admittedly, we have not examined the effects of these two drugs administered by inhalation.

Our results are at variance with those obtained by O'Connell et al. (1994) showing that in healthy human volunteers oral or inhaled clonidine had no effects on capsaicin-induced cough. The reasons for these discrepancies remain unclear. However, differences in the animal species and in the type of preparation may have played a role. For instance, in the human studies cough was induced by means of inhalation of capsaicin and mechanical stimulation was not tested. It could be suggested that the cough-related afferent fibers activated by capsaicin are conveyed to brainstem regions not responsive to *α*_2_-adrenergic receptor agonists and/or that functionally active *α*_2_-adrenergic receptors are not present on sensory nerves in the normal human airways.

Both nociception and cough share similar features (e.g., Barnes 2007; Canning 2009; Ji et al. 2009; Mutolo et al. 2012; Lavinka and Dong 2013). Thus, it is not surprising that our results are consistent with previous findings on the involvement of *α*_2_-adrenergic receptors in central antinociceptive effects (see Chan et al. 2010 for reviews). It is well known that effective analgesia is provoked by the activation of spinal *α*_2_-adrenergic receptors by noradrenaline or other agonists in animals and humans (Eisenach et al. 1996; Philipp et al. 2002; Pertovaara 2006). Similarly, an involvement of MAPK pathways has been demonstrated in the mechanisms underlying the activation of primary afferent neurons implicated in pain transmission (e.g., Ji et al. 2009) as well as in cNTS sensory mechanisms underlying the regulation of the cough reflex (Mutolo et al. 2012).

### Effects of clonidine and tizanidine on the sneeze reflex

The specificity of the *α*_2_-adrenergic receptors as well as of the brainstem injected areas in cough regulation is corroborated by the lack of clonidine and tizanidine effects on the sneeze reflex. The question remains on which mechanisms have a role in the modulation of nasotrigeminal reflex responses by inputs from nasal and vagal afferents that converge on the cNTS. As discussed in our previous reports (Mutolo et al. 2009, 2012), the absence of changes in sneeze reflex responses could be reasonably attributed to the fact that the primary site of central nasal projections is the sensory complex of the trigeminal nerve (Lucier and Egizii 1986; Wallois et al. 1995). Probably, NTS may constitute only a location where subsidiary regulatory functions are executed (Macron et al. 1994; Dutschmann et al. 1998; Plevkova et al. 2009). In this context, it is appropriate to mention that previous studies (Mutolo et al. 2009) have shown that blockade of ionotropic glutamate receptors at the level of the cVRG attenuates or suppresses the expiratory thrusts of sneezing without affecting the inspiratory preparatory bursts, thus indicating that cVRG neurons are simply an output system for the expiratory component of the reflex. The absence of significant effects of *α*_2_-adrenergic receptor agonists on the sneeze reflex indicates that not only peripheral but also central afferent pathways of this reflex, including those converging on cVRG neurons, are separated from the corresponding pathways of the cough reflex and do not require an involvement of *α*_2_-adrenergic receptors.

### Conclusions

As already discussed, present results are at variance with previous findings obtained in guinea pigs and humans (O'Connell et al. 1994). In the light of the relatively strong downregulation of the cough reflex observed in the rabbit following clonidine and tizanidine administration, we believe that this subject deserves further investigation in humans and in different animal species to ascertain the antitussive effects and to provide new insights into the site and mechanism of action of *α*_2_-adrenergic receptor agonists. Present findings also encourage further studies to develop novel antitussive *α*_2_-adrenergic compounds active on different *α*_2_-adrenergic receptor subtypes possibly devoid of strong side effects and, in particular, hypotensive responses, in an attempt to provide enhancements in treatments of cough.
